# A case of neurofibromatosis type 1 with neurofibromatosis type 1-related and neurofibromatosis type 1-unrelated tumors: a case report

**DOI:** 10.1186/s13256-025-05599-z

**Published:** 2025-10-13

**Authors:** Tabea I. Hartung, Qais Karimi, Lan Kluwe, Said C. Farschtschi

**Affiliations:** 1 Department of Neurology, University Medical Center Hamburg-Eppendorf, Martinistr. 52, 20246 Hamburg, Germany; 2 Department of Maxillofacial Surgery, University Medical Center Hamburg-Eppendorf, Hamburg, Germany

**Keywords:** Neurofibromatosis type 1, NF1, Rare tumor spectrum, NF1-unrelated tumors

## Abstract

**Background:**

Neurofibromatosis type 1 is an autosomal dominantly inherited disorder caused by pathogenic variants in the *neurofibromatosis type 1* gene, resulting in a predisposition to multiple tumors. Typical neurofibromatosis type 1-associated tumors include cutaneous and plexiform neurofibromas, optic pathway gliomas, breast cancer, and malignant peripheral nerve sheath tumors. The aim of the study was to report a rare case with multiple neurofibromatosis type 1-unrelated tumors in addition to neurofibromatosis type 1-related ones.

**Case presentation:**

A 36-year-old Caucasian female patient with neurofibromatosis type 1 had a rare tumor spectrum including ganglioglioma, malignant teratoma, pheochromocytoma, vestibular schwannoma, and meningioma in addition to typical neurofibromatosis type 1 tumors such as cutaneous and plexiform neurofibromas. A pathogenic variant in the *neurofibromatosis type 1* gene (c.2446C > T, p.Arg816*) was identified in blood-derived DNA while no pathogenic variant was found in the *neurofibromatosis type 2* gene.

**Conclusion:**

Though rare, multiple neurofibromatosis type 1-unrelated tumors can develop in neurofibromatosis type 1 patients, which demands attention and interdisciplinary management. All related genes should be screened for potential pathogenic variants.

## Background

Neurofibromatosis type 1 (NF1) is one of the most common autosomal dominantly tumor predisposition syndromes, with an incidence of approximately 1 in 2600 to 1 in 3000 newborns [1]. NF1 is caused by pathogenic variants in the *NF1* gene on chromosome 17, which encodes the tumor suppressor protein neurofibromin [2]. This protein plays a key role in negatively regulating the RAS/mitogen-activated protein kinase signaling pathway, and its loss of function leads to tumor predisposition in patients with NF1. NF1 is characterized by various clinical manifestations, such as café-au-lait macules, bilateral skinfold freckling, Lisch nodules, or bone abnormalities, and is associated with benign and malignant tumor entities [2–5].

The hallmark of NF1 is multiple benign neurofibromas on nerve sheath [3]. Cutaneous neurofibromas typically develop during puberty and increase in size and number throughout adulthood [6–8]. Plexiform neurofibromas are also typical NF1 tumors and carry a lifetime risk of 8–13% of transforming into a malignant peripheral nerve sheath tumor [9–11]. Low-grade gliomas, particularly optic pathway gliomas, are the most common central nervous system tumors associated with NF1 [12]. Pheochromocytomas are less frequent (0.1–5.7%) in patients with NF1 but need to be considered in the diagnostic workup by unexplained hypertensive blood pressures, as these tumors are characterized by excessive catecholamine production, leading to hypertension and other systemic symptoms [12–14]. Furthermore, there is at least a fivefold increased risk of breast cancer in women with NF1 under 50 years of age [15–18]. Spinal cord gangliogliomas are extremely rare tumors in general population but have been reported in patients with NF1, suggesting possible association [19].

This study presents a case of a female patient with NF1 with an untypical tumor spectrum.

## Case presentation

In 2022, a 36-year-old Caucasian female patient first presented at the Neurofibromatosis Outpatient Clinic of the University Medical Center Hamburg-Eppendorf in Hamburg, Germany. She presented with a multiple tumor manifestations (Table 1) and typical clinical manifestations, ensuring the diagnosis of NF1, which was genetically confirmed by identifying a pathogenic variant in the *NF1* gene: c.2446C > T, p.(Arg816*). The patient had no family history of NF1 or other tumor predisposition syndromes.

**Table 1:** Rare tumor spectrum reported in this case

Tumor	Location		Age at diagnosis	Diagnose procedure	Treatment	
Uncommon NF1-related tumors						
Ganglioglioma	Right parieto-occipital		24	MRI, histopathology	Resection + post-hemorrhage treatment	
Pheochromocytoma	Right adrenal gland		28	High blood pressure, adrenal mass by MRI, high catecholamines,	Retroperitoneoscopic adrenalectomy; postoperative colostomy and later reversal	
NF1-unrelated tumors						
Benign ovarian tumor		Right ovary	22	Solid mass by ultrasound, histopathology confirmation		Total resection
Malignant teratoma		Left ovary	36	14-cm multicystic mass by pelvic MRI, histology confirmed immature malignant teratoma (pT1c2G3)		Laparotomy with adnexectomy and adhesiolysis + adjuvant chemotherapy with cisplatin and etoposide (three cycles)
Teratoma recurrence		Left pelvis	37	1.3-cm tumor by pelvic MRI		Hysterectomy and oophorectomy
Vestibular schwannoma		Right	38	Whole-body MRI including cranial MRI		Regular imaging and hearing controls
Meningioma		Sagittal falx cerebri	38	Whole-body MRI including cranial MRI		Regular clinical and imaging control

### Typical NF1-related manifestations

The patient presented with multiple café-au-lait macules, bilateral axillary and inguinal freckling, cutaneous neurofibromas, and plexiform neurofibromas. Skeletal abnormalities, such as thoracolumbar scoliosis, splay foot, and leg length discrepancy, were also documented.

### Uncommon NF1-related tumors.

At the age of 24 years, an asymptomatic ganglioglioma in the right parieto-occipital region was identified by magnetic resonance imaging (MRI), which was followed by surgical excision in the same year. At the age of 28 years, markedly hypertensive blood pressure was noted and MRI revealed a right-sided pheochromocytoma. Therapeutic management included a retroperitoneoscopicadrenalectomy. However, the procedure resulted in an iatrogenic bowel injury requiring a colostomy, which was later reversed with stomal closure surgery. Follow-up examinations revealed no tumor recurrence.

### NF1-unrelated tumors

At the age of 22, the patient was diagnosed with a benign, right-sided ovarian tumor, which was surgically resected. At the age of 36 years, the patient complained about menstrual irregularities, prompting further investigation. Pelvic MRI revealed a large, multicystic mass measuring up to 13.6 cm, likely originating from the left ovary. Histologically, an immature malignant teratoma of the left ovary was diagnosed, followed by a laparotomy with left adnexectomy and adhesiolysis. The teratoma was classified as high-grade lesion and staged as pT1c2G3. After complete surgical resection, the patient underwent adjuvant chemotherapy with three cycles of cisplatin and etoposide, and 1 year later, follow-up MRI revealed tumor recurrence along the left pelvic wall. Hysterectomy and oophorectomy were performed. No tumor recurrence was detected in further follow-up examinations.

At the age of 38 years, whole-body MRI including cranial MRI was performed to search for NF1-related internal tumors, and revealed a right-sided intrameatal vestibular schwannoma and a falx meningioma. As these are typical manifestations of *NF2*-related schwannomatosis, we conducted a genetic testing of the *NF2* gene on blood-derived DNA, which was negative. However, a mosaic form of *NF2*-pathogenic variant could not be excluded. No tumor specimen was available for the genetic testing. Extensive genetics testing, including whole-exome sequencing, was not performed in this case.

## Discussion and conclusion

This case report illustrates a rare and complex tumor spectrum in a patient with NF1, including both uncommon NF1-related tumors, such as ganglioglioma and pheochromocytoma. and NF1-unrelated tumors, such as malignant teratoma, vestibular schwannoma. and meningioma.

Vestibular schwannomas in patients with NF1 is exceedingly rare [20]. Meningiomas are occasionally observed in patients with NF1, but do not show a significantly increased frequency compared with the general population [21]. Meningiomas and vestibular schwannomas are common sporadic tumors, and both are typical tumors in the condition of *NF2*-related schwannomatosis [22]. However, in this case, the diagnostic criteria for a *NF2*-related schwannomatosis were not met, though a mosaic form cannot be excluded. No pathogenic variant of the *NF2* gene was found in the blood-derived DNA. It would be very interesting to test the tumors. However, no specimen is available since the tumors have not yet been resected [22, 23].

A malignant teratoma in a patient with NF1 adds another layer of complexity to this case. While teratomas are rare in the general population, their occurrence in patients with NF1 is even more infrequent. Mature teratomas are typically benign, whereas immature teratomas are malignant [24, 25]. To date, there are two cases of teratomas reported in patients with NF1. A testicular teratoma was previously reported in a 23-year-old man with NF1 [26]. Additionally, there is one case report of a congenital teratoma in a patient with NF1 [27]. Again, tumor-based genetic testing could provide valuable insights as to the potential role of the inactivation of the *NF1* gene in malignant teratoma.

We cannot fully exclude other causes leading to some NF1-unrelated tumors. For example, chemotherapy for the teratoma could be the cause for the secondary development of benign schwannomas. This correlation has been described for radiotherapy in the past [28, 29].

The unusual tumor spectrum in this case does not seem to be specifically linked to the pathogenic variant in the *NF1* gene: c.2446C > T, p.(Arg816*), since this variant is a recurrent one and has been found in 17 unrelated cases in our patient collection, who, on the basis of our database, do not have such unusual tumor spectrum.

Clinical manifestations of NF1 are readily complex with a long list of features and parameters demanding consideration and care. This may lead to reduced attention for other symptoms that are unrelated to NF1 and therefore not on the checklist. However, NF1 condition does not protect the patients from developing unrelated manifestations, and therefore any unrelated abnormalities also need to be further examined.

The occurrence of ganglioglioma, pheochromocytoma, malignant teratoma, vestibular schwannoma, and meningioma in one single patient with NF1 represents an exceptionally rare and complex condition. Extended genetic testing for all relevant genes, in addition to the *NF1* gene, on blood- and tumor-derived DNA should be considered. Adequate management of such complex and atypical cases needs multidisciplinary teamwork of medical professionals.

## Data Availability

The datasets used and/or analyzed during the current study are available from the corresponding author on reasonable request.

## Abbreviations

NF1: Neurofibromatosis type 1
MRI: Magnetic resonance imaging
